# Development of highly sensitive electrochemical biosensor for the detection of hydroquinone using a FAD functionalized fluorapatite/SWCNT hybrid composite modified electrode

**DOI:** 10.1039/d5ra01981h

**Published:** 2025-05-29

**Authors:** Mehtab Singh Sidhu, Gokul Sridharan, Dhanraj Ganapathy, Raji Atchudan, Sandeep Arya, Surendra H. Mahadevegowda, Ashok K. Sundramoorthy

**Affiliations:** a Centre for Nano-Biosensors, Department of Prosthodontics, Saveetha Dental College and Hospitals, Saveetha Institute of Medical and Technical Sciences 162, Poonamallee High Road, Velappanchavadi Chennai 600077 Tamil Nadu India ashok.sundramoorthy@gmail.com; b School of Chemical Engineering, Yeungnam University Gyeongsan 38541 Republic of Korea; c Department of Physics, University of Jammu, Jammu Jammu and Kashmir 180006 India; d Department of Chemistry, School of Sciences, National Institute of Technology Andhra Pradesh Tadepalligudem 534101 Andhra Pradesh India

## Abstract

Hydroquinone (HQ) is a toxic and carcinogenic substance commonly used in cosmetics, pharmaceuticals, and various industrial applications. In this study, we report the synthesis of a novel composite material consisting of flavin adenine dinucleotide (FAD) functionalized fluorapatite (FA) combined with single-walled carbon nanotubes (SWCNTs) (FAD/FA/SWCNT). This composite was prepared through a mechanochemical method using dimethyl sulfoxide (DMSO) as the dispersing solvent. The resulting composite was then applied on to a glassy carbon electrode (GCE) for the electrochemical detection of hydroquinone (HQ). The structural morphology and phase of the nanocomposite were characterized using field emission scanning electron microscopy (FESEM), X-ray diffraction (XRD), and UV-visible spectroscopy. Electrochemical techniques, including cyclic voltammetry and amperometry, were employed to evaluate the electrochemical performance of the surface-modified electrode for hydroquinone (HQ) detection. The FAD/FA/SWCNT biosensor exhibited a redox peak at −0.45 V in a 0.1 M phosphate buffer solution (PBS, pH 7.4) under blank conditions. The composite demonstrated remarkable catalytic activity, showing an increased current signal in response to HQ detection. The sensor displayed a linear response range from 0.005 μM to 258.2 μM for HQ, with a limit of detection (LOD) of 2.70 nM. Furthermore, the FAD/FA/SWCNT biosensor successfully detected HQ in spiked water samples, achieving high recovery rates between 100.4% and 103.0%, highlighting its potential for real-world applications.

## Abstract 2

Hydroquinone (HQ) is a toxic and carcinogenic substance commonly used in cosmetics, pharmaceuticals, and various industrial applications.

## Graphical abstract

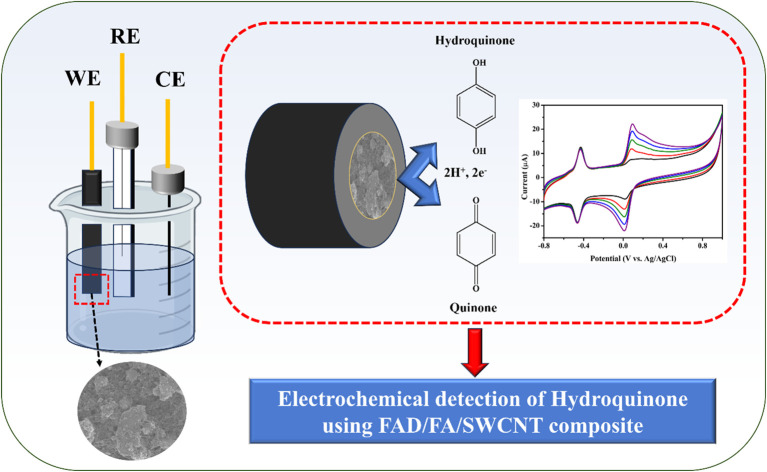

## 1. Introduction

Hydroquinone (HQ) is widely used across various industries, including food production, home goods, pharmaceuticals, textiles, and paper bleaching. Beyond its cytotoxic effects, HQ plays significant roles in processes such as immune cell activation, vascular remodeling, stomatal closure signaling, and the regulation of various biological activities. Additionally, HQ serves as a byproduct of enzymatic reactions catalyzed by oxidases like glutamate, lysine, d-amino acid, lactate, and cholesterol, making it essential for these biochemical processes. Maintaining appropriate levels of HQ is critical for proper cellular function and intracellular signaling. Excessive HQ can lead to aging, biological damage, neurodegeneration, and even cancer,^1^ highlighting the importance of monitoring its levels in living organisms.

Several analytical methods have been developed to detect HQ, including spectrophotometry,^2^ chemiluminescence,^3^ titrimetric,^4^ and fluorimetry.^5^ However, these techniques often require expensive equipment, labor-intensive sample preparation, skilled operators, and long analysis times. In contrast, electroanalytical biosensors offer a promising alternative, providing benefits such as interference-free, highly selective, and sensitive detection, simplified sample preparation, no need for expert personnel, suitability for field applications, and real-time data acquisition.^6–10^

Fluorapatite (FA) and hydroxyapatite (HA) are versatile biomaterials that belong to the same calcium phosphate family, with chemical formulas of Ca_5_(PO_4_)_3_F and Ca_5_(PO_4_)_3_(OH), respectively.^11–14^ Hydroxyapatites have been widely used in various electrochemical sensing applications, including the detection of l-tyrosine, hydrazine, bisphenol A, uric acid, kidney injury molecules (KIM), cadmium, and lead.^15–21^ Due to its multiple adsorption sites and unique three-dimensional structure, HA has attracted considerable attention in the sensor field.^22,23^ Additionally, the surface charge of HA can be influenced by its morphological and structural characteristics, such as the carbonate content, surface Ca/P ratio, and the presence of acidic phosphate groups like HPO_4_^2−^.^24^

To our knowledge, there is limited literature supporting the use of FA in sensing applications. Therefore, we synthesized FA using a traditional hydrothermal method and created a composite with single-walled carbon nanotubes (SWCNTs) for HQ detection. Flavin adenine dinucleotide (FAD), a cofactor of riboflavin (vitamin B2), is known for its unique fluorescence properties, which arise from its isoalloxazine ring structure and the associated adenine moiety that acts as a quencher. FAD exists in three different conformations: stacked, unstacked, and partially stacked.^25^ The structure, fluorescence characteristics, and stability of FAD can vary depending on the pH.^26^ Due to its distinctive properties, FAD has numerous applications, including in imaging and catalysis.^27^ Moreover, FAD-modified electrodes have been explored for a variety of electrochemical biosensor applications due to their efficient electron transport capabilities.^28^ However, directly immobilizing FAD on bare glassy carbon electrodes (GCE) or other noble metal electrodes has proven challenging due to its size and instability.^29^

FAD has been used as a catalyst in many biosensor applications over the years. For instance, Kumar *et al.* developed a hydrogen peroxide sensor using an FAD/chitosan/CNT/ITO electrode, achieving a limit of detection (LOD) of 1 μmol L^−1^.^27^ More recently, Medrades *et al.* used FAD to functionalize gold nanoparticles for dopamine detection in human urine, reporting an LOD of 0.525 μmol L^−1^, and highlighted its catalytic role in sensor applications.^30^ Electrochemical sensors have also been explored for detecting a wide range of small molecules using various nanomaterials and hybrid composite with various architectures.^10,31–37^ Numerous electrochemical sensors have been developed for the detection of HQ, but significant challenges remain in improving sensitivity, selectivity, stability, and detection range. Peng *et al.* proposed an electrochemical HQ sensor based on a MoS_2_/RGO hybrid composite, achieving an exceptionally low detection limit of 0.3 nM, the lowest reported to date. However, its linear detection range (1–9 nM) is relatively narrow.^38^ While subsequent studies have aimed to extend this linear range, these efforts often result in a trade-off, either compromising the detection limit or failing to significantly enhance overall sensor performance.^38–46^ Therefore, our study aims to develop a novel electrode based on a FAD/FA/SWCNT hybrid composite, which we believe could serve as a highly sensitive tool for HQ detection, offering a broader linear range suitable for real-world samples. To our knowledge, no prior research has explored this composite for HQ detection, nor has any work achieved such a low limit of detection with such a wide linear range in earlier studies.

## 2. Experimental

### 2.1 Materials and reagents

Flavin adenine dinucleotide disodium salt hydrate (FAD) was purchased from the high purity laboratory chemicals Pvt. Ltd. Disodium ethylene diamine tetra acetic acid (EDTA), calcium nitrate, diammonium hydrogen phosphate, dimethyl sulfoxide (DMSO), sodium fluoride, and potassium chloride (KCl) were acquired from Sigma-Aldrich, India. Single-walled carbon nanotubes (SWCNTs) were obtained from Carbon Solutions, Inc., (Columbia Ave, USA). Potassium ferricyanide K_3_[Fe(CN)_6_], potassium ferrocyanide K_4_[Fe(CN)_6_], and ammonium hydroxide (NH_4_OH) (25% extra pure AR) were obtained from Sisco Research Laboratories (SRL), Pvt. Ltd. India. All the experiments were conducted using a 0.1 M phosphate buffer solution (PBS, pH 7.4), which was prepared by utilizing sodium phosphate dibasic heptahydrate (Na_2_HPO_4_·7H_2_O) and sodium dihydrogen phosphate monohydrate (H_2_NaPO_4_·H_2_O) obtained from Spectrochem Pvt, Ltd., India. All the solutions were prepared using deionized water (DI H_2_O) (18.2 MΩ cm) at a temperature of 25 ± 2 °C.

### 2.2 Synthesis of fluorapatite (FA)

A clear solution was obtained by dissolving 5 mM EDTA in 50 mL of deionized water (DI H_2_O) and stirred it thoroughly. Subsequently, a solution containing 5 mM calcium nitrate (Ca (NO_3_)_2_·4H_2_O) and 3 mM diammonium hydrogen phosphate ((NH_4_)_2_ HPO_4_) was prepared using the EDTA solution. 0.1 M HNO_3_ and 0.1 M NaOH were added to the resultant mixture to bring its pH down to 3.7. After that, 1.5 mM NaF was added to the mixture and stirred for 10 minutes. The mixture was then transferred to a hydrothermal vessel lined with Teflon. The hydrothermal treatment was conducted at 160 °C for 8 hours. The solution was centrifuged at 8000 rpm for 10 minutes multiple times until it reached a neutral pH. Following several decantation's, the solution was dried overnight at 60 °C, thoroughly ground with a mortar, and stored in a vial for future use.^47^

### 2.3 Mechanochemical preparation of FAD/FA/SWCNT composite

**Scheme 1:**
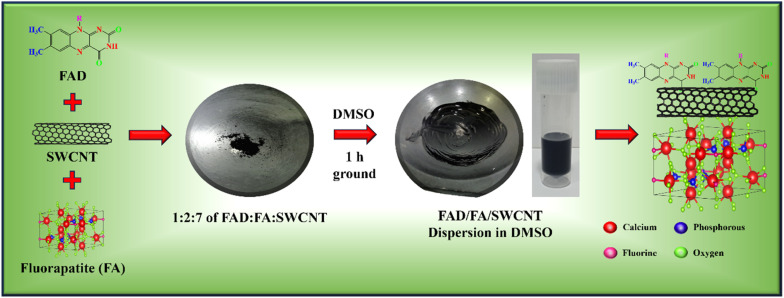
Schematic illustration for the preparation of the FAD/FA/SWCNT composite.

To prepare a homogeneous paste, FAD, FA, and commercial SWCNTs were combined in amounts of 1 mg, 2 mg, and 7 mg, respectively. A few drops of DMSO were added to the mixture, and it was thoroughly ground for 1 hour using a mortar and pestle. To achieve a well-dispersed, viscous FAD/FA/SWCNT composite paste, 2 mL of DMSO was added, and the mixture was sonicated to ensure a uniform dilution. The final composite was then stored in a vial for future use (Scheme 1). For comparison, the same procedure was repeated with the FA/SWCNT composite, without FAD.

### 2.4 Fabrication of FAD/FA/SWCNT modified electrode

A GCE (diameter ∼3 mm) was polished using alumina powder (0.05-micron) until a mirror-like, reflective surface was obtained. After polishing, the GCE was left outside to dry naturally. It was sonicated for five minutes after diluting 100 μL of FAD/FA/SWCNT dispersion in 2 mL of DMSO. Next, a 7 μL FAD/FA/SWCNT dispersion was drop cast onto a GCE and dried at 50 °C for 10 minutes. An electrode that had been modified with a FAD/FA/SWCNT composite was used to perform the electrochemical detection of HQ in 0.1 M PBS. To eliminate dissolved oxygen and dissolve the analyte uniformly, nitrogen gas was used to purge into the PBS throughout each electrochemical experiment, which was conducted at room temperature.^33,48^

### 2.5 Characterizations

To verify the crystalline structure of the FAD/FA/SWCNT composite, an X-ray diffractometer (D8-Advance, BRUKER, Mannheim, Germany) was employed to obtain an X-ray diffraction (XRD) pattern in the 20–90° range. The FAD/FA/SWCNT dispersion was drop cast onto the silicon wafer and dried for an entire night at 100 °C to prepare the sample. Removing hydroxide groups changed the material's phase from amorphous to crystalline. Cu Kα radiation with a wavelength of 1.5406 Å was utilized to get XRD patterns at a voltage of 40 kV and a current of 15 mA. The scan rate used to generate the XRD pattern was 10° min^−1^. To compare changes and confirm the crystallographic pattern of FA, standard reference was plotted using VESTA software and the crystallographic open database (COD ID – 1010996).^49^ UV-visible (UV-vis) spectra were gathered with a UV-Jasco spectrophotometer for FAD/FA/SWCNT composite dispersed in DMSO solvent. Using high-resolution scanning electron microscopy (HR-SEM), the composite's surface morphology was identified. An instrument operating at 1.10 kV (QUANTA 200F HRSEM) was used for HR-SEM analysis. For this purpose, FAD/FA/SWCNT film was prepared on an aluminum foil.

### 2.6 Sensor preparation and electrochemical measurements

We used a CHI 760E electrochemical workstation from CH Instruments (Austin, TX, USA) to conduct electrochemical experiments. A standard three-electrode setup was utilized: a platinum wire was used as the counter electrode, a GCE that had been modified with FAD/FA/SWCNT to act as the working electrode, and an Ag/AgCl reference electrode that was submerged in 1 M KCl. The GCE underwent preliminary preparations before being used. It was sonicated in deionized water (DI H_2_O) after alumina powder (Al_2_O_3_) (0.05-micron) was used to polish it to a mirror-like surface. After that, the GCE underwent ten cycles of potential scanning, ranging from 0.5 to 1.0 V, as part of an electrochemical treatment in 0.1 M H_2_SO_4_. Following this procedure, 7 μL of FAD/FA/SWCNT dispersion was cast on the GCE surface and then dried at 50 °C. Following a brief air-drying at room temperature, the FAD/FA/SWCNT/GCE was submerged in to DI H_2_O to remove any unbound catalyst material from the electrode surface. This procedure was repeated to apply a second coating of FAD/FA/SWCNT. Finally, the prepared FAD/FA/SWCNT/GCE electrode was used to detect HQ in 0.1 M PBS.

## 3. Results and discussion

### 3.1 SEM and UV-vis spectroscopy

**Fig. 1:**
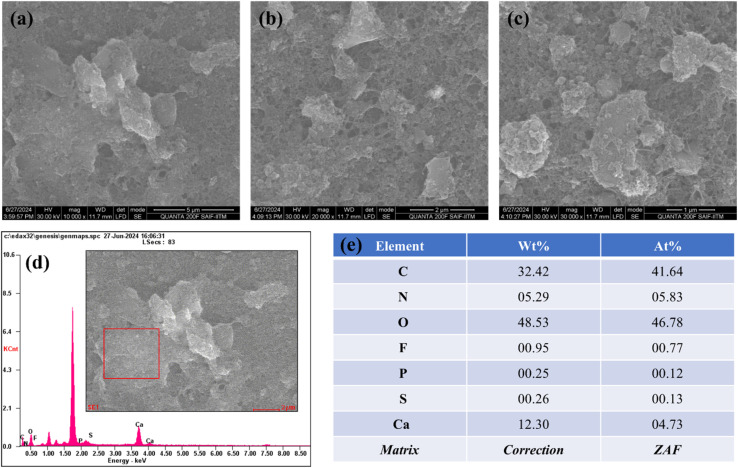
HRSEM images of FAD/FA/SWCNT at 5 μm (a), 2 μm (b), and 1 μm (c) resolutions. (d) Energy-dispersive spectrum (EDS) (inset: a scanned area for the analysis) and (e) the weight ratio of elements presents in FAD/FA/SWCNT composite.

The morphological analysis obtained from HRSEM is shown in Fig. 1a–c, illustrating the prepared FAD/FA/SWCNT composite at three different magnifications. The observed morphology is a result of the synthesis method, with the mechanochemical synthesis technique producing a well-defined structure that exhibits a large surface area. The composite appears as clusters of sheets distributed across an extensive area. This structure is capable of delivering strong electro-catalytic responses. The addition of DMSO facilitates proper dispersion, while the mechanochemical method contributes to achieving a large surface area. Fig. 1d presents the EDS spectrum of the elements present in the selected area of the composite (shown in the inset), from which the elemental weight ratios are derived (Fig. 1e). This characteristic is crucial for sensor applications and offers substantial advantages in nanomaterial synthesis. With a uniform distribution and optimal particle size, the material is well-suited for further electrochemical studies and analyte detection.

**Fig. 2:**
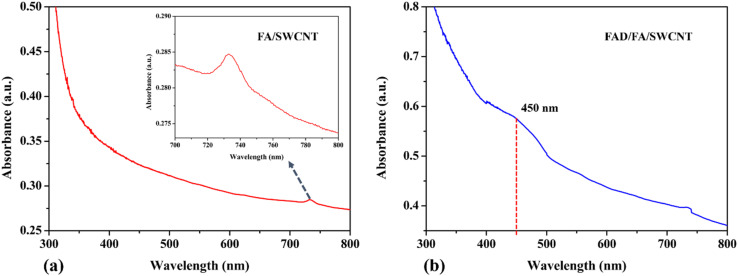
UV-visible spectra of FA/SWCNT (a) and FAD/FA/SWCNT (b).

Fig. 2a displays the UV-vis spectrum of an FA/SWCNT composite dissolved in DMSO, where the prominent peak at 732 nm corresponds to the inter-band transition associated with the metallic properties of SWCNTs.^50^ It is worth mentioning that the absorption bands of SWCNTs were not clearly observed, which was due to the bundling of the nanotubes. As noted in previous reports, FA does not show any significant absorbance peaks within the 300 to 800 nm range.^51^ It is important to note that the presence of FA cannot be confirmed based solely on the UV-vis spectrum. Fig. 2b shows the UV-vis spectrum of the FAD/FA/SWCNT composite, where the absorbance peak at 450 nm indicates the presence of FAD, which contains an isoalloxazine ring. This peak is associated with a π–π* transition linked to its oxidized state; a result consistent with previous studies.^52^

### 3.2 X-ray diffraction (XRD) analysis

**Fig. 3:**
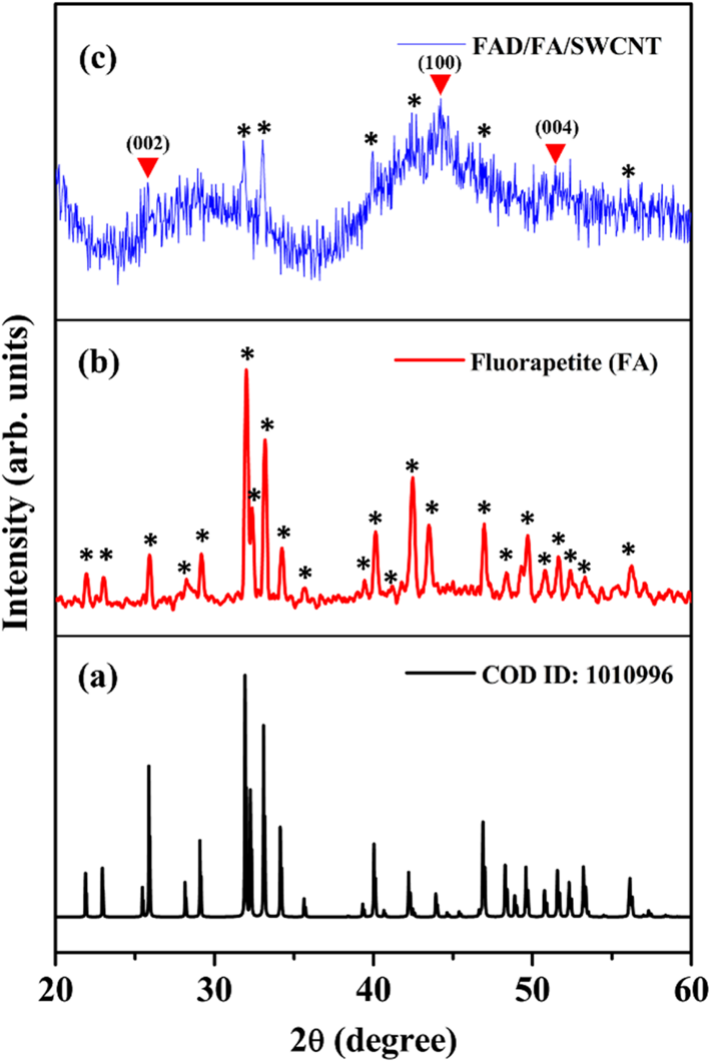
XRD patterns of (a) FA (reference data), (b) as prepared FA, and (c) FAD/FA/SWCNT composite.

The crystalline structure of the FAD/FA/SWCNT composite was analyzed using XRD, and the results were compared with the XRD data for standard FA (Fig. 3). Fig. 3a presents the XRD pattern of pure FA, which closely matches that of the as-synthesized FA. In Fig. 3b, the black star symbol indicates the 2*θ* values corresponding to the diffracted angles of FA. The hexagonal crystal structure of FA is characterized by 2*θ* values at 21.90°, 23.03°, 25.93°, 28.25°, 29.16°, 32.0°, 32.40°, 33.17°, 34.23°, 35.64°, 39.44°, 40.15°, 41.17°, 42.48°, 43.50°, 46.99°, 48.36°, 49.68°, 50.79°, 51.64°, 52.36°, 53.27°, and 56.21°, which correspond to specific orientations of (200), (111), (002), (102), (210), (121), (112), (300), (202), (301), (122), (130), (103), (302), (113), (222), (132), (213), (231), (140), (303), (004), and (223) (COD ID: 1010996).^49^Fig. 3c displays the diffraction pattern of the FAD/FA/SWCNT composite, where the red triangle symbol indicates peaks corresponding to pristine SWCNTs at 2*θ* values of 25.77°, 44.19°, and 51.45°, which align with the (002), (100), and (004) planes of graphitic carbon, respectively.^53–55^ A peak at 44.19° represents disordered carbon, while a smaller peak at 25.77° corresponds to the crystalline carbon's *d*-spacing.^6,54^ The black star symbol identifies the presence of FA in the composite, and by comparing the 2*θ* values at 31.84°, 33.03°, 39.96°, 42.40°, 46.90°, and 56.15° with those in Fig. 3b, we observe a slight shift of the peaks to lower angles. This shift is likely attributed to the bonding interaction between FA and SWCNTs, which leads to an increase in the interlayer spacing.

### 3.3 Electrochemical impedance spectroscopy (EIS)

**Fig. 4:**
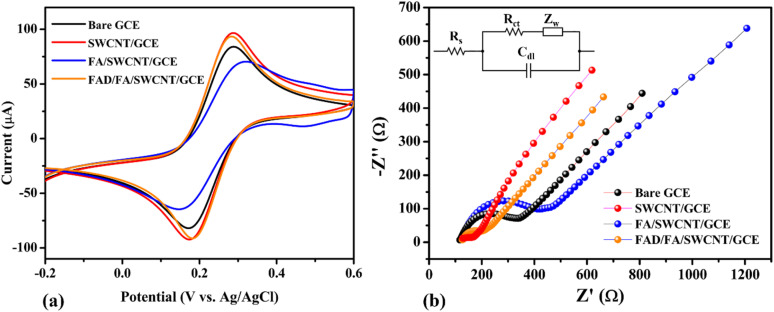
Cyclic voltammograms (CVs) (a) and Nyquist plots (b) of bare GCE (black), SWCNT/GCE (red), FA/SWCNT/GCE (blue), and FAD/FA/SWCNT/GCE (orange) (Inset: Randles circuit).

The electrochemical behavior of the FAD/FA/SWCNT hybrid composite modified GCE was evaluated using cyclic voltammetry (CV) and EIS in a 0.1 M KCl solution containing 5 mM [Fe(CN)_6_]^3−/4−^. These measurements were compared against those of bare and other modified GCEs to assess the efficiency of electron transfer. Fig. 4a shows the CV responses at a scan rate of 50 mV s^−1^. Among all tested electrodes, the FAD/FA/SWCNT/GCE (orange curve) demonstrated the highest redox peak current (*I*_p_ = 94.36 μA) and the smallest peak-to-peak separation (Δ*E*_p_ = 102 mV), indicating superior electron transfer kinetics. In contrast, the FA/SWCNT/GCE (blue) exhibited a lower peak current of 70.80 μA and a broader Δ*E*_p_ of 174 mV, while the bare GCE (black) recorded an *I*_p_ of 84.81 μA with a Δ*E*_p_ of 117 mV. Interestingly, the pristine SWCNT/GCE (red) showed a slightly higher current (*I*_p_ = 97.38 μA) and a comparable Δ*E*_p_ of 115 mV, attributable to the excellent electrical conductivity of SWCNTs. However, the incorporation of FA, despite its inherently lower conductivity, provided distinct benefits. FA contributes a large surface area and forms strong π–π interactions with SWCNTs, which enhance the immobilization of FAD molecules. The synergistic integration of FAD, FA, and SWCNTs not only facilitated more efficient electron transfer but also improved the overall stability and sensitivity of the modified sensor.^56^

Nyquist plots for the electrolyte solution of 5 mM [Fe(CN)_6_]^3−/4−^ + 0.1 M KCl are shown for the bare GCE (black), SWCNT/GCE (red), FA/SWCNT/GCE (blue), and FAD/FA/SWCNT/GCE (orange) in Fig. 4b (with the inset showing the Randles circuit). The small semicircle with a tail suggests a diffusion-controlled process. Due to the high conductivity of SWCNTs, the Nyquist plot for SWCNT/GCE (25.23 Ω) shows a lower *R*_ct_ value compared to the bare GCE (169.8 Ω), which can be attributed to the metallic characteristics of SWCNTs, as evidenced by the UV-vis spectrum of SWCNTs.^50^ In contrast, the *R*_ct_ value for FA/SWCNT/GCE (251.2 Ω) was higher than that of both bare GCE (169.8 Ω) and SWCNT/GCE (25.23 Ω), indicating that the inclusion of FA increased the *R*_ct_, likely due to FA's large bandgap and bulk structure.^57^ After FAD deposition on the FA/SWCNT/GCE surface, the *R*_ct_ decreased to 66.7 Ω (Table 1), suggesting that the electron transport of the electrochemical probe at the modified electrode was minimally affected by the adsorption of FAD. The changes in *R*_ct_ values for each electrode modification, along with their underlying explanations, are supported by literature references, as indicated by the differences observed in the bare GCE (black), SWCNT/GCE (red), FA/SWCNT/GCE (blue), and FAD/FA/SWCNT/GCE (orange).^27^

**Table 1:** EIS parameters and their respective values for bare and other modified GCEs

Sample	*R*_s_ (Ω)	*R*_ct_ (Ω)	*C*_dl_ (F)	*Z*_W_ (Ω s^−1/2^)
Bare GCE	123.4	169.8	8.67 × 10^−7^	5.97 × 10^−4^
SWCNT/GCE	124.2	25.23	1.95 × 10^−7^	6.83 × 10^−4^
FA/SWCNT/GCE	131.9	251.2	3.56 × 10^−7^	3.75 × 10^−4^
FAD/FA/SWCNT/GCE	127.9	66.7	8.18 × 10^−7^	6.47 × 10^−4^

### 3.4 Electrochemical properties of FAD/FA/SWCNT/GCE

**Scheme 2:**
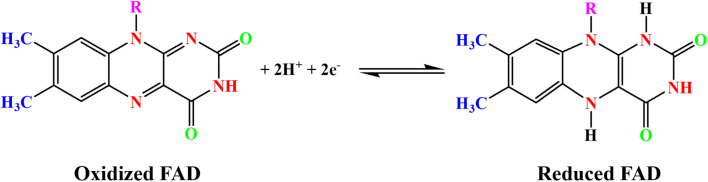
FAD redox process at pH 7.4 in 0.1 M PBS.

The electrochemical performance of the fabricated biosensor, consisting of FAD/FA/SWCNT/GCE, was evaluated using CV. The analysis was conducted within a potential range of −0.8 V to 1.0 V at a scan rate of 50 mV s^−1^ in a 10 mL 0.1 M PBS (pH 7.4, N_2_ saturated). The results confirmed that the FA/SWCNT composite effectively preserved the natural state of FAD on the GCE surface and facilitated the efficient loading of FAD molecules. In contrast, the inclusion of FAD resulted in a distinct redox peak for the FAD/FA/SWCNT/GCE, as shown in Scheme 2.^58^

**Fig. 5:**
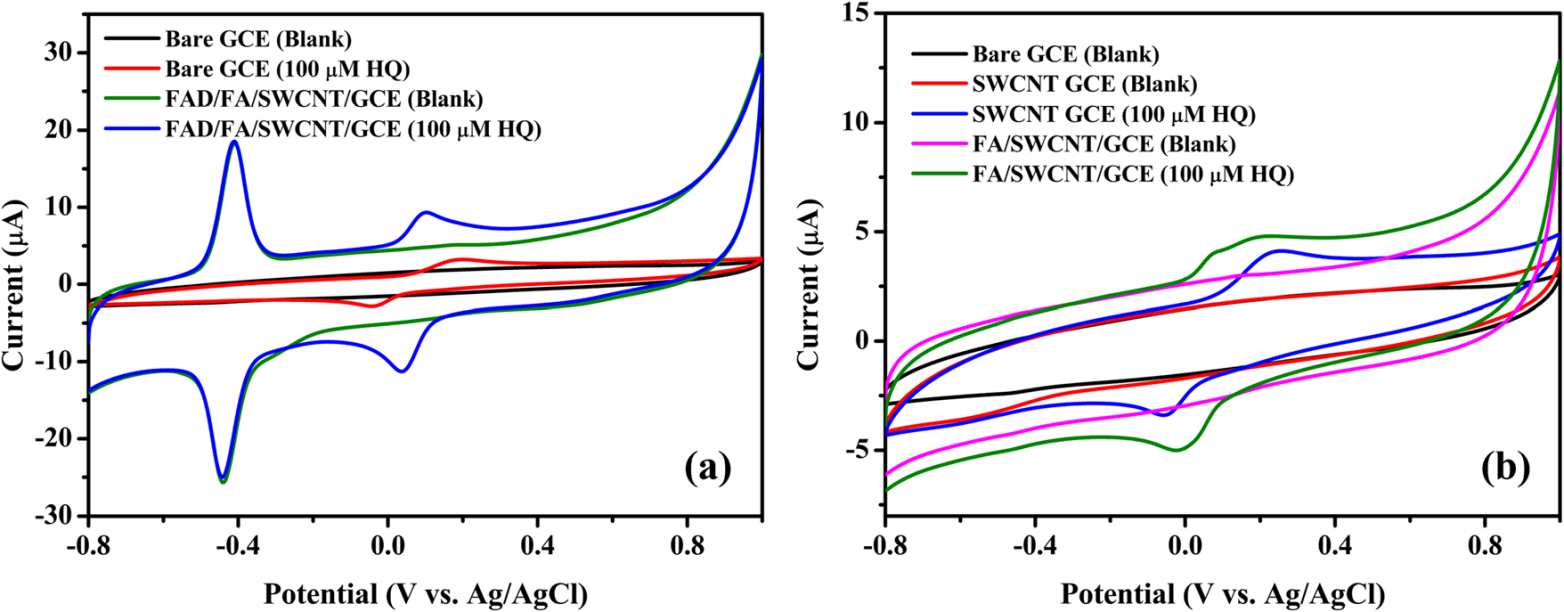
(a) CVs of bare GCE and FAD/FA/SWCNT/GCE in 0.1 M PBS (scan rate = 50 mV s^−1^) with and without 100 μM HQ. (b) CVs of SWCNT/GCE and FA/SWCNT/GCE (b) in 0.1 M PBS in the absence and presence of 100 μM HQ (for comparison, CVs of bare GCE is also given in the absence of HQ).

As depicted in Fig. 5a, both anodic and cathodic scans revealed an oxidation peak (*E*_pa_) at −0.4290 V and a reduction peak (*E*_pc_) at −0.4625 V for FAD, respectively. The formal potential (*E*°′ = [*E*_pa_ + *E*_pc_]/2) for FAD was calculated to be −0.45 V, which is consistent with results from other studies on FAD-modified electrodes.^59^ For the detection of 100 μM HQ, the FAD/FA/SWCNT-modified GCE exhibited an anodic current of 9.59 μA at 0.09 V, which is 2.3 times higher than the current observed for the bare GCE (3.22 μA at 0.19 V). The electrochemical redox reaction of HQ is reversible. As shown in Fig. 5b, neither the SWCNT/GCE nor the FA/SWCNT/GCE displayed a redox peak at −0.45 V. For 100 μM HQ detection, the SWCNT/GCE exhibited a slight shift to a higher potential (0.24 V, 4.23 μA), while the FA/SWCNT/GCE showed a shift to a lower potential (0.19 V, 4.84 μA). This potential shift to a lower value is attributed to the presence of FA and the π–π stacking interaction with SWCNTs, which enhances the catalytic properties toward HQ sensing. The charge transfer coefficient (*α*) is typically assumed to be 0.5,^60^ and so the number of electrons transferred during the HQ redox reaction was calculated as two using the relevant equation.^61–64^

1 (*E*_p_ − *E*_p/2_) = Δ*E*_p_ = 1.857*RT*/*αnF*

where *E*_p/2_ is the potential at half the peak current, *R* is the gas constant (8.314 J K^−1^mol^−1^), *T* is the room temperature (298 K), and *F* is the Faraday constant (96 485 C mol^−1^).

The results demonstrated that the overall catalytic effect is attributed to the combined contributions of the various materials in the composite (Table 2). Specifically, the lower potential observed is due to the presence of FA, while the enhanced sensitivity toward HQ is attributed to the FAD component. Additionally, the stability of the sensor is significantly improved by the inclusion of SWCNTs.

**Table 2:** Electrochemical oxidation details of HQ on various electrodes

Electrode	*E*_pa_ (V)	*E*_pc_ (V)	*I*_pa_ (μA)	*I*_pc_ (μA)
Bare GCE	0.19	−0.04	3.22	−2.82
SWCNT/GCE	0.24	−0.05	4.23	−3.42
FA/SWCNT/GCE	0.19	−0.02	4.84	−5.04
FAD/FA/SWCNT/GCE	0.09	0.03	9.59	−11.49

### 3.5 Linear determination of HQ

**Fig. 6:**
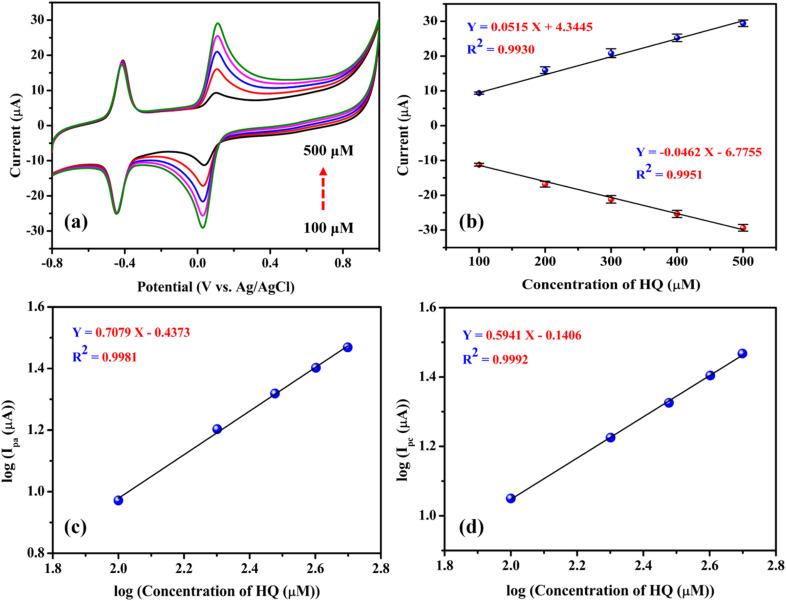
(a) CVs of FAD/FA/SWCNT/GCE recorded for linear concentrations of hydroquinone (100, 200, 300, 400, 500 μM) in 0.1 M PBS (pH 7.4, scan rate = 50 mV s^−1^). (b) A calibration plot of HQ was recorded between the concentrations of HQ (μM) *vs.* peak current (μA). (c) Log of anodic peak current (*I*_pa_) (μA) *vs.* log of the concentration of HQ (μM), and (d) log of cathodic peak current (*I*_pc_) (μA) (d) *vs.* log of the concentration of HQ (μM).

**Scheme 3:**
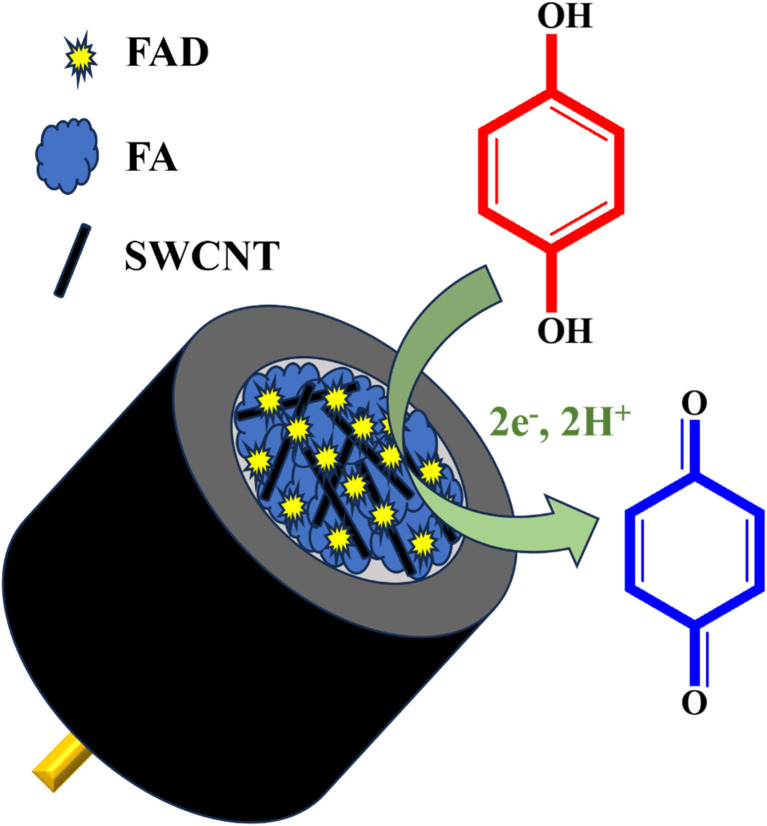
Electrochemical redox reaction of HQ to *p*-benzoquinone on FAD/FA/SWCNT/GCE.

Fig. 6a presents the CV responses of the FAD/FA/SWCNT-modified GCE with varying concentrations of HQ in N_2_-saturated 0.1 M PBS (pH 7.4) at a scan rate of 50 mV s^−1^. The oxidation and reduction peak potentials are approximately 0.08 V and 0.02 V, respectively. The electrochemical biosensor, based on the FAD/FA/SWCNT-modified GCE, shows a gradual increase in the oxidation peak current as HQ concentration increases from 100 μM to 500 μM, indicating the sensor's ability to detect higher concentrations of HQ. These CV results highlight the strong electrocatalytic performance of the FAD/FA/SWCNT-modified GCE in the HQ redox process (Scheme 3). A linear relationship between the redox peak currents (μA) and HQ concentration (μM) was established from 100 μM to 500 μM with a maximum standard deviation (SD) of 2.21% (anodic peak current) and 1.87% (cathodic peak current). The linear regression equations were *I*_pa_ = 0.0515 (HQ/μM) + 4.3445 and *I*_pc_ = −0.0462 (HQ/μM) – 6.7755. The correlation coefficients values were (*R*^2^) 0.9930 and 0.9951, respectively (Fig. 6b), demonstrated the excellent electrochemical activity of the FAD/FA/SWCNT-modified GCE toward the HQ redox reaction. Further, Fig. 6c and d display the calibration plots of the logarithmic anodic and cathodic peak currents against the log of HQ concentration. The regression equations, *I*_pa_ (log(μA)) = 0.7079 (log(μM)) − 0.4373 (*R*^2^ = 0.9981) and *I*_pc_ (log(μA)) = 0.5941 (log(μM)) − 0.1406 (*R*^2^ = 0.9992), were derived from these plots. The results indicate that the electrochemical oxidation of HQ on the FAD/FA/SWCNT-modified GCE follows a first-order kinetics.

### 3.6 Effect of pH

**Fig. 7:**
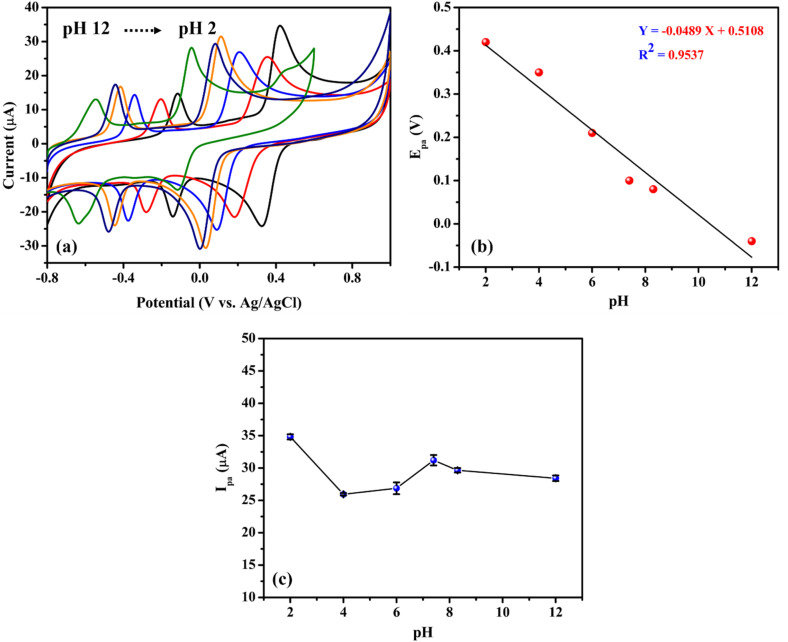
(a) CVs of FAD/FA/SWCNT/GCE recorded with 500 μM hydroquinone in 0.1 M PBS of different pH 2, 4, 6, 7.4, 8.3, 12 at a scan rate of 50 mV s^−1^. (b) A linear plot was made between different pH solution *vs.* anodic peak potentials (*E*_pa_) (V), and (c) between different pH solution *vs.* anodic peak current (*I*_pa_) (μA).

The electrochemical activity of FAD/FA/SWCNT/GCE was examined in different pH buffer solutions (from 2 to 12) with 500 μM HQ. CV was performed to characterize the impact of varying solution pH values, as seen in Fig. 7a. The electrochemical redox reaction of HQ is significantly influenced by the pH of the solution because protons participate in the electrochemical oxidation process. A negative shift in peak potential was seen when the pH of the solution increased, suggesting that the HQ oxidation at the FAD/FA/SWCNT is a pH-dependent process. The linear regression equation that follows was used to characterize the connection between pH and *E*_pa_ (Fig. 7b), *E*_pa_ (V) = −0.0489pH + 0.5108 (*R*^2^ = 0.9537). Over the pH range of 2.0 to 12.0, the slope was determined to be −48.9 mV pH^−1^, which is near the theoretical value of −59 mV pH^−1^ and demonstrates the two-electron transfer process coupled with two protons (Scheme 3). Since the pH values of mineral and tap water are almost equal to 7, a 7.4 pH of 0.1 M PBS was selected as the ideal pH value for the analysis. Usually, the phenolic hydroxyl groups of HQ can be effectively adsorbed onto the functional groups of FAD/FA/SWCNT hybrid composite, maybe to the phosphate or oxygen-containing sites of FA. This proton-coupled electron transfer leads to the formation of benzoquinone, completing the oxidation process.

### 3.7 Effect of scan rate

**Fig. 8:**
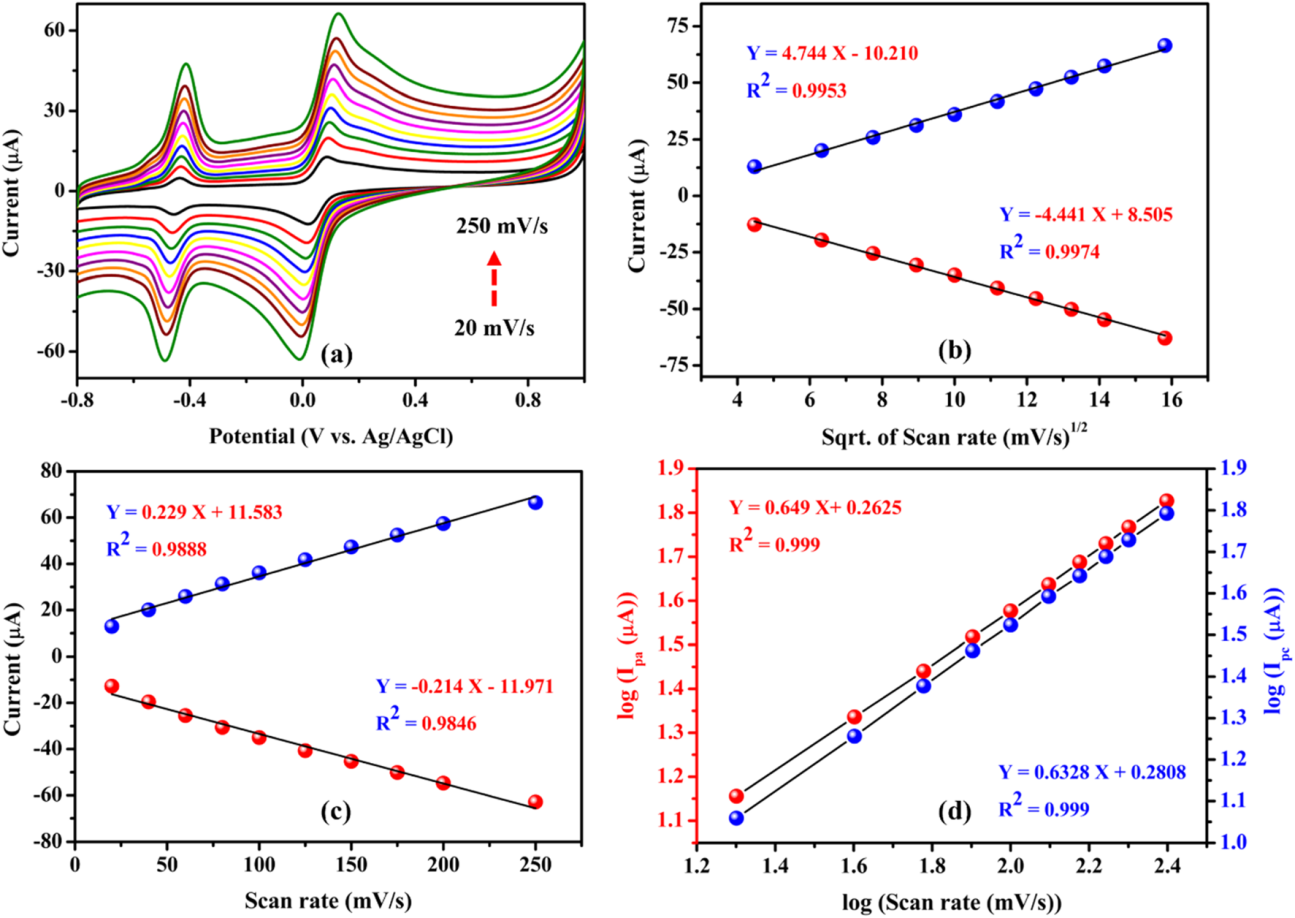
Cyclic voltammograms of FAD/FA/SWCNT recorded at different scan rates (20, 40, 60, 80, 100, 125, 150, 175, 200, 250 mV s^−1^) with 500 μM hydroquinone in 0.1 M PBS (pH 7.4) (a). A linear plot was made between *I*_pa_ and *I*_pc_ (μA) *vs.* sqrt. of the scan rate (mV s^−1^)^1/2^ (b). A linear plot for *I*_pa_ and *I*_pc_ (μA) *vs.* scan rate (mV s^−1^) (c). A linear plot was made between the log of scan rate (mV s^−1^) *vs.* log of (*I*_pa_ and *I*_pc_ (μA)) (d).

To investigate the effect of scan rate on the performance of the FAD/FA/SWCNT-modified GCE, cyclic voltammetry was performed at varying scan rates (20 to 250 mV s^−1^) with a fixed concentration of hydroquinone (500 μM) in N_2_-purged 0.1 M PBS (pH 7.4). The results showed a linear increase in both the oxidation and reduction peak currents, accompanied by a linear shift in the anodic and cathodic peak potentials. This shift in the redox peak potential is attributed to the influence of the diffusion layer thickness, which is inversely proportional to the scan rate^65–67^ (Fig. 8a).

Fig. 8b illustrates that the redox peak currents (*I*_pa_ and *I*_pc_) were linearly proportional to the square root of the scan rate (*ν*^1^/^2^) over the range of 20 to 250 mV s^−1^. The corresponding linear regression equations, *I*_pa_ (μA) = 4.744 (mV s^−1^)^1/2^ − 10.210 (*R*^2^ = 0.9953) and *I*_pc_ (μA) = −4.441 (mV s^−1^)^1/2^ + 8.505 (*R*^2^ = 0.9974), indicate that the redox reaction of HQ is a diffusion-controlled process.^68^ However, Fig. 8c demonstrates the redox peak currents (*I*_pa_ and *I*_pc_) to the scan rate (*ν*), which is less linear than the square root of the scan rate (*ν*^1^/^2^), with regression equations, *I*_pa_ (μA) = 0.229 (mV s^−1^) + 11.583 (*R*^2^ = 0.9888) and *I*_pc_ (μA) = −0.214 (mV s^−1^) – 11.971 (*R*^2^ = 0.9846), suggesting that the redox reaction of HQ is not controlled by adsorption process.^65^ Additionally, Fig. 8d shows the linear plot for the log of peak current *versus* the log of scan rate. The corresponding regression equations, log(*I*_pa_ (μA)) = 0.649 log(mV s^−1^) + 0.2625 (*R*^2^ = 0.999) and log(*I*_pc_ (μA)) = 0.632 log(mV s^−1^) + 0.2808 (*R*^2^ = 0.999), reveal slope values between 0.5 and 1 for both the anodic and cathodic curves, which is closer to 0.5. This data indicated that the electrode kinetics of the system involve more diffusion-controlled processes than adsorption-controlled for the HQ redox reaction on the FAD/FA/SWCNT-modified GCE.^69–73^

### 3.8 Interference, repeatability, and reproducibility studies

**Fig. 9:**
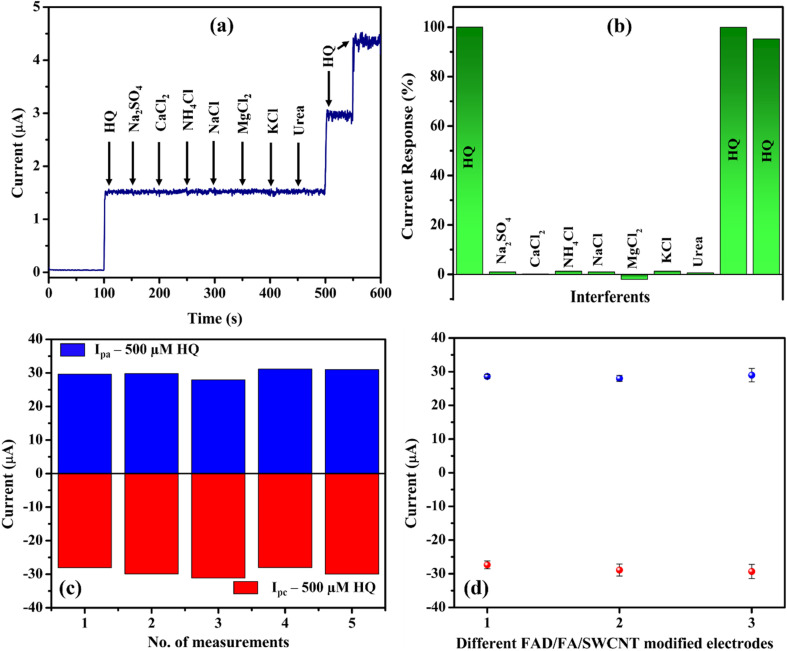
(a) Amperogram of FAD/FA/SWCNT/GCE in 0.1 M PBS detecting 10 μM HQ in the presence of interferents (Na_2_SO_4_, CaCl_2_, NH_4_Cl, NaCl, MgCl_2,_ KCl and urea; 100 μM concentration each) at an applied potential of +0.09 V (stirring rate = 720 rpm). (b) Histogram of current responses *vs.* interferents. Repetitive CV response of FAD/FA/SWCNT/GCE with the same electrode (c) and CV response of FAD/FA/SWCNT/GCE for three different electrodes (d) detecting 500 μM HQ in 0.1 M PBS (pH 7.4, scan rate of 50 mV s^−1^).

Amperogram was measured in PBS (0.1 M, pH 7.4) with 10 μM HQ along with the added interferents such as sodium sulphate, calcium chloride, ammonium chloride, sodium chloride, magnesium chloride, potassium chloride, and urea at a concentration 10-fold higher than that of HQ (Fig. 9a and b). These cations, anions, and molecules were commonly present in tap water and mineral water, so we have chosen them for the selectivity analysis. The HQ oxidation peak remains constant even after the addition of various interfering compounds. These findings showed that these interferents did not generate any oxidation currents, so that the FAD/FA/SWCNT hybrid composite modified GCE had good selectivity for HQ. It was possibly because the oxidation potential of interferents was observed at different potentials, apart from the oxidation potential of HQ. Based on the above findings, FAD/FA/SWCNT hybrid composite can be employed to detect HQ in water samples with high selectivity. To analyse the repeatability of the sensor, the one-time modified FAD/FA/SWCNT/GCE was tested for 500 μM HQ in 0.1 M PBS (Fig. 9c), indicating an excellent accuracy of the fabricated FAD/FA/SWCNT sensor for the detection of HQ. Three different FAD/FA/SWCNT modified GCEs were prepared, and their redox peak current responses for 500 μM HQ were found to be similar to each other as shown in Fig. 9d. These results indicated the acceptable reproducibility of the proposed HQ sensor. The fabricated electrochemical sensor possesses a good anti-interference properties, good repeatability, and reproducibility.

### 3.9 Amperometry and stability studies

**Fig. 10:**
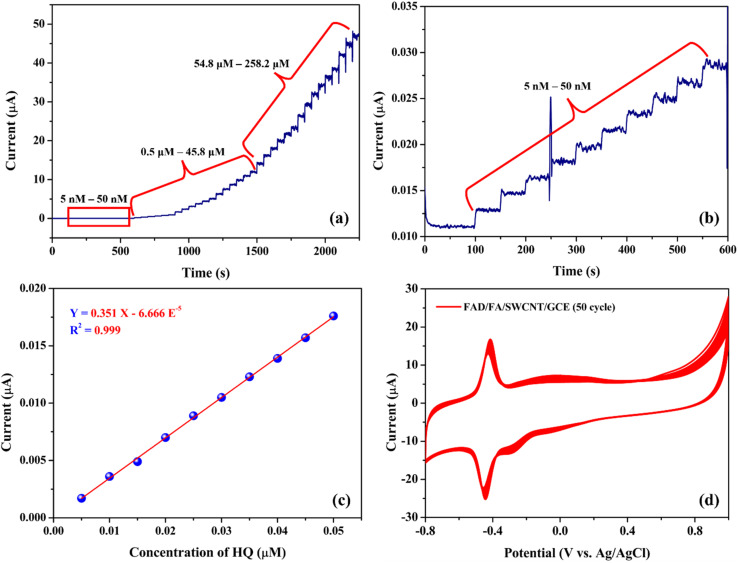
(a and b) Amperometric current responses of FAD/FA/SWCNT/GCE to successive addition of HQ in PBS (0.1 M, pH 7.4). Applied potential = 0.09 V. (c) Calibration graph of hydroquinone. (d) CV responses of FAD/FA/SWCNT in 0.1 M PBS (pH 7.4) for 50 cycles at a scan rate of 50 mV s^−1^.

Amperometry (*i*–*t*) is a highly effective technique for assessing the performance of the biosensor, especially for detecting trace levels, due to its excellent sensitivity.^32^Fig. 10a shows the amperometric responses of the FAD/FA/SWCNT/GCE for HQ oxidation at an applied potential of 0.09 V. The electrolyte used in this experiment was 20 mL of 0.1 M PBS (pH 7.4). The FAD/FA/SWCNT/GCE exhibited a linear response in current following each addition of HQ, ranging from 0.005 to 258.2 μM. A calibration plot of HQ concentration *versus* oxidation current revealed a linear relationship between 5 and 50 nM (Fig. 10b), with a high correlation coefficient (*R*^2^ = 0.999) (Fig. 10c). The sensors response time was four seconds (Fig. 10b).

Fig. 10d demonstrates the stability of the FAD/FA/SWCNT-modified GCE, showing consistent performance over 50 continuous cycles of cyclic voltammetry in 0.1 M PBS (pH 7.4). The LOD for the proposed HQ biosensor was calculated to be 0.0027 μM, using the formula provided (eqn (2)).^6^2
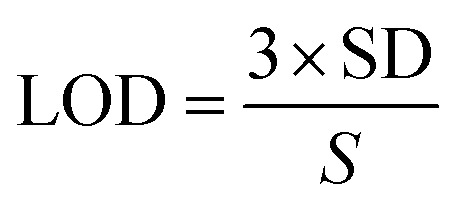


The standard deviation (SD) of the blank was found to be 3.176 × 10^−4^ μA, while the slope (*S*) of the calibration graph was 0.351 μA μM^−1^. A detailed comparison of the electrochemical performance of the FAD/FA/SWCNT-modified GCE with previously reported electrodes for HQ detection is summarized in Table 3. This comparison highlights that the FAD/FA/SWCNT-modified GCE exhibits excellent response properties, making it a highly suitable platform for HQ detection.

**Table 3:** The analytical performance of various modified electrodes reported for HQ sensing^a^

S. no	Electrode	LOD (nM)	Linear range (μM)	Method	Ref.
1	MoS_2_/RGO	0.3	0.001–0.009	DPV	38
2	Laser-induced graphene/Al NPs/PET	56	0.1–300	DPV	39
3	EP(ALN)MCNTPE	174	0.2–4	DPV	40
4	Cu SAs/N-CSs	330	1 – 324	DPV	41
5	Ga_2_O_3_-doped ZnO (Ga_2_O_3_·ZnO)	63	1–11070	Amp	42
6	FeWO_4_/SnO_2_/Nf	1.3	0.01–50	DPV	74
7	RGO/MWCNT	400	3.0–150.0	DPV	44
8	COF/CPE	310	1–2000	DPV	45
9	MWCNT-COOH/CTF-1	120	2–280 μM	DPV	46
10	FAD/FA/SWCNT	2.70	0.005–258.2	Amp	This work

a Al NPs – aluminum nanoparticles, PET – polyethylene terephthalate, EP(ALN)MCNTPE – electrochemically polymerized l-alanine modified carbon nanotube paste electrode, Ga_2_O_3_·ZnO – gallium oxide doped zinc oxide, FeWO_4_/SnO_2_/Nf – iron tungstate doped tin oxide nanocomposite Nafion, RGO/MWCNT: reduced graphene oxide/multiwall carbon nanotube, COF/CPE – covalent organic framework modified carbon paste electrode, MoS_2_ – molybdenum disulfide, MWCNT-COOH/CTF-1 – carboxylated multi-walled carbon nanotubes and covalent triazine framework.

The superior electrocatalytic activity of the proposed biosensor can be attributed to the synergistic effect of FAD, FA, and SWCNT, which collectively enhance the interaction with HQ molecules, providing a more effective catalyst than any individual component. Additionally, the high conductivity and large surface area of FAD-functionalized FA/SWCNT contribute significantly to the remarkable electrocatalytic performance observed. By addressing the defects in FA/SWCNT, FAD introduces numerous anchor sites, ensuring sustained high catalytic activity for HQ sensing. In conclusion, the FAD/FA/SWCNT-modified GCE proves to be a highly effective and promising sensor for the electrochemical detection of HQ.

### 3.10 Real sample analysis

**Fig. 11:**
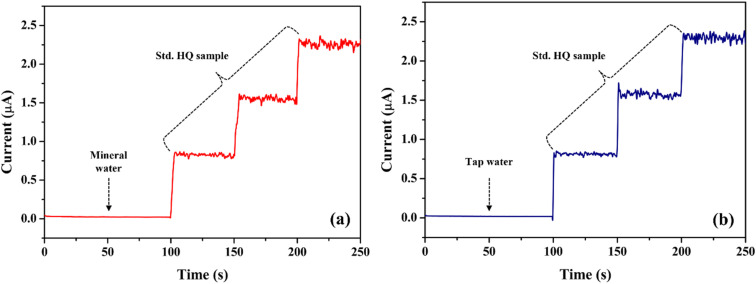
Amperometry detection of the HQ in spiked mineral water (a), and tap water (b) samples. Applied potential = 0.09 V.

The electrochemical performance of the FAD/FA/SWCNT-modified GCE was evaluated using real samples, specifically mineral water and tap water, collected from the Saveetha Dental College (SDC) campus. For the mineral water, 50 μL of the sample was added to 20 mL of 0.1 M PBS, and HQ was spiked in three consecutive concentrations (2.5, 5, and 7.5 μM) at 50-second intervals using the standard addition method (Fig. 11a). The same procedure was applied for tap water spiked sample (Fig. 11b). The results demonstrated excellent detection of HQ, with recovery rates ranging from 100.4% to 103.0%, indicating the high accuracy and reliability of the proposed biosensor for HQ detection in real-world samples (Table 4). These findings highlight that the proposed HQ biosensor offers exceptional versatility, making it suitable for both laboratory standard samples and real-world applications with consistent and reliable performance.

**Table 4:** Determination of HQ in real-world samples using the standard addition method

Sample	HQ Added (μM)	HQ found (μM)	Recovery (%)
Mineral water	2.50	2.55	102.0
	5.00	5.03	100.6
	7.50	7.60	101.3
Tap water	2.50	2.51	100.4
	5.00	5.13	102.6
	7.50	7.73	103.0

## 4. Conclusion

In this study, FA was utilized for the first time in biosensor development, and a novel FAD-functionalized FA/SWCNT nanocomposite was successfully synthesized and characterized. Spectroscopic and morphological analyses (UV-vis, XRD, HRSEM, and EDAX) confirmed the successful integration of FAD into the composite and revealed the characteristic hexagonal crystalline structure of FA. The resulting FAD/FA/SWCNT-modified GCE displayed outstanding electrocatalytic activity toward the oxidation of HQ, with a low anodic peak potential of 0.09 V at 7.4 pH. The biosensor achieved a wide linear detection range from 0.005 μM to 258.2 μM HQ and an impressive detection limit of 2.70 nM, demonstrating excellent sensitivity. Real sample analysis further validated the sensor's reliability, achieving high recovery rates between 100.4% and 103.0%. These findings highlight the synergistic role of FAD as a redox mediator and fluorapatite as a biocompatible, high-surface-area support material, establishing the FAD/FA/SWCNT composite as a powerful platform for electrochemical biosensing. As a future advancement, this sensing strategy could be integrated into lab-on-chip biosensor devices for miniaturized, real-time, and point-of-care detection of hydroquinone. This would enhance portability, reduce sample/reagent volumes, and enable rapid environmental or clinical diagnostics in real-world samples.

## Data availability

The data will be made available upon a reasonable request.

## Author contributions

Mehtab Singh Sidhu: writing – original draft. Gokul Sridharan: methodology, formal analysis, conceptualization, writing – original draft. Dhanraj Ganapathy: validation, data curation. Raji Atchudan: resources, review and validation. Sandeep Arya: editing and validation, formal analysis. Surendra H. Mahadevegowda: editing and validation, formal analysis. Ashok K. Sundramoorthy: writing – review & editing, funding acquisition, supervision, project administration.

## Conflicts of interest

The authors declare that they have no known competing financial interests or personal relationships that could have appeared to influence the work reported in this paper.
